# Deep Active Learning for Automatic Segmentation of Maxillary Sinus Lesions Using a Convolutional Neural Network

**DOI:** 10.3390/diagnostics11040688

**Published:** 2021-04-12

**Authors:** Seok-Ki Jung, Ho-Kyung Lim, Seungjun Lee, Yongwon Cho, In-Seok Song

**Affiliations:** 1 Department of Orthodontics, Korea University Guro Hospital, Seoul 08308, Korea; jgosggg@korea.ac.kr; 2 Department of Oral and Maxillofacial Surgery, Korea University Guro Hospital, Seoul 08308, Korea; ungassi@korea.ac.kr; 3 Department of Oral and Maxillofacial Surgery, Korea University Anam Hospital, Seoul 02841, Korea; brianlee877@gmail.com; 4 Department of Radiology, Korea University Anam Hospital, Seoul 02841, Korea

**Keywords:** active learning, maxillary sinusitis, convolutional neural network, deep learning, segmentation

## Abstract

The aim of this study was to segment the maxillary sinus into the maxillary bone, air, and lesion, and to evaluate its accuracy by comparing and analyzing the results performed by the experts. We randomly selected 83 cases of deep active learning. Our active learning framework consists of three steps. This framework adds new volumes per step to improve the performance of the model with limited training datasets, while inferring automatically using the model trained in the previous step. We determined the effect of active learning on cone-beam computed tomography (CBCT) volumes of dental with our customized 3D nnU-Net in all three steps. The dice similarity coefficients (DSCs) at each stage of air were 0.920 ± 0.17, 0.925 ± 0.16, and 0.930 ± 0.16, respectively. The DSCs at each stage of the lesion were 0.770 ± 0.18, 0.750 ± 0.19, and 0.760 ± 0.18, respectively. The time consumed by the convolutional neural network (CNN) assisted and manually modified segmentation decreased by approximately 493.2 s for 30 scans in the second step, and by approximately 362.7 s for 76 scans in the last step. In conclusion, this study demonstrates that a deep active learning framework can alleviate annotation efforts and costs by efficiently training on limited CBCT datasets.

## 1. Introduction

Deep learning technology is advancing daily. Previously, it was only used in some areas such as image processing; however, artificial intelligence (AI) technology using deep learning has been used in various fields. In particular, deep learning technology using convolutional neural networks (CNNs) has excellent performance in analyzing image information [1,2]. This is going beyond object detection to find a specific object in an image, object classifications to classify which object it is, and this continues to develop into object segmentation, a technology that finds and separates the area of a specific object. Among them, object segmentation is the most difficult technique [3].

AI technology that analyzes and evaluates images is creating a lot of synergy in the medical field. In particular, this makes a significant contribution to the field of diagnosis [4]. Owing to the characteristics of medical fields, determining whether or not there is a specific disease using X-ray radiographs, computed tomography (CT), and magnetic resonance imaging (MRI) data is the most active field in which artificial intelligence technology is used. Analysis of whether there is a lesion, such as cancer, or what kind of disease the lesion is being performed [5,6]. Similar studies have been conducted in the dental field in recent years [7,8]. Likewise, the dental field is a field where many X-rays and CTs are taken, and the evaluation and diagnosis of the image is essential [9]. It would be nice to be able to obtain the help of highly skilled experts every time, but there is a lot of possibilities that a general practitioner may miss when it comes to difficult diseases. For this, if AI can screen for and inform a specific disease, the general practitioner can be alert to the diagnosis, and if necessary, it can be referred to a higher-level hospital or specialist. Accordingly, many previous studies have analyzed panoramic images to analyze tooth decay and periodontal disease and to detect changes in alveolar bone [10,11]. In addition, lesions for malignant diseases, such as ameloblastoma, are easily detected and not missed so that the patient’s disease can be detected early [12].

In the dental field, not only diseases related to the teeth, but also the maxillary sinus is the subject of much interest [13,14]. The maxillary sinus is also an important part of the dental field, such as maxillary molar tooth disease, which causes maxillary sinusitis; if the maxillary molar implant has insufficient bone, maxillary sinus elevation is performed and bone grafts are performed. Accordingly, it is very helpful to accurately diagnose, analyze, and evaluate maxillary sinus diseases. In a two-dimensional panoramic picture, the maxillary sinus area is distorted and overlapped by the vertebrae, which is difficult to evaluate [14]. CT data, which are 3D images, are necessary for the accurate evaluation of the maxillary sinus. Deep learning analysis of 3D images is a much more difficult area than the analysis of 2D images. It is a complex area that needs to be reconstructed and evaluated again after analysis of the 2D slice image. The maxillary sinus is connected to various sinuses, such as the nasal cavity, ethmoid sinus, and frontal sinus, and is adjacent to the orbit and skull in the upper direction; therefore, it is very difficult to separate. Thus, it is even more difficult to segment the disease in the maxillary sinus.

Therefore, we studied a technique for segmenting maxillary sinus diseases using deep learning technology using 3D cone-beam computed tomography (CBCT) data. Segmentation has developed significantly with the development of CNN technology, but it is difficult to obtain sufficient labeled data for training in medical data. In this study, a customized 3D U-Net capable of active learning was used to increase training efficiency with limited data and reduce labeling efforts. This technology improves performance in an organic and dynamic way in which a person evaluates and corrects the result determined by artificial intelligence, and the artificial intelligence reflects and learns it again. The aim of this study was to segment the maxillary sinus into the maxillary bone, air, and lesion, and to evaluate its accuracy by comparing and analyzing the results performed by experts. We also determined whether active learning could improve segmentation accuracy and labeling efficiency.

## 2. Materials and Methods

### 2.1. Datasets and Pre-Processing

We used CBCT datasets (103 patients-internal and 20 patients-external) of consecutively patients with various sinuses that were confirmed between January 2018 and May 2020. All CBCT were acquired on the KAVO 3D Exam, Model 17–19 (Imaging Sciences International, Hatfield, PA, USA) for internal data and CS 9300 (Carestream Dental, GA, USA) for external data. In each scan, both bilateral maxillary sinuses were entirely visible. The exclusion criteria were when the radiograph quality was poor due to artifacts, there was an abnormality in the maxillary sinus or a history of surgery in the maxillary sinus.

Maxillary sinus segmentation was performed using CBCT. We randomly selected 83 patients for deep active learning (Table 1). For training, tuning, and testing, all datasets were split into 70:10:20 ratios. The ground truth of 40 cases for the first step with 20-internal Korea University Anam Hospital (KUAH) and 20 external Korea University Ansan Hospital (KUANH) scans for testing were verified by an expert reviewer using the AVIEW software, version 1.0.3 (Coreline Software, Seoul, Korea). In the second and last steps, 64 cases were used for active learning with limited data.

All input volumes were resized to 320 × 320 pixels with intensity normalization using the mean and standard deviation of the pixel on volumes. Third-order spline interpolation was performed by resampling each label separately. Aggressive data augmentation was used with the batch generator framework, involving gamma correction augmentation, random scaling, random rotations, random elastic deformations, and mirroring [15].

### 2.2. Training Architecture

**Figure 1:**
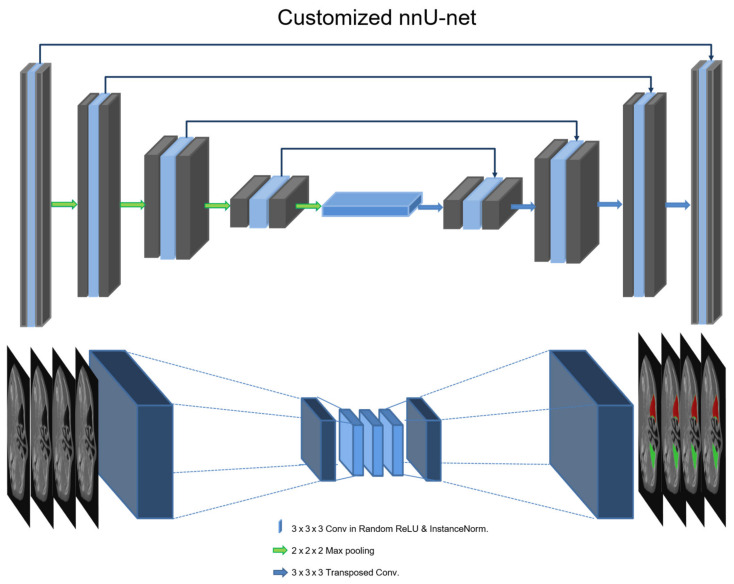
Deep learning architecture of the customized 3D U-Net in the nnU-Net.

The 3D U-Net of nnU-Net was used for maxillary sinus segmentation, including air and lesions in CBCT [16,17]. This architecture (customized 3D U-Net) is shown in Figure 1. The architecture comprises an encoder and a decoder network with transposed convolutional layers for backward operations. The left side reduces the dimensionality of the input, and the right side recovers the original dimensionality. The architecture involves 30 convolutional filters in the first layer and max pooling (2 × 2 × 2). The encoder network is similar to a conventional convolution neural network (CNN), which results in the reduction of spatial information and a loss of localization accuracy. In pixel-wise segmentation, both spatial and semantic information are important for training and testing medical images or volumes. The decoder of U-Net exploits deconvolution with a skip connection to maintain spatial information using semantic information from the low vertex. In this study, we replaced the leaky rectified linear unit (ReLU) activation functions with random ReLU of the original 3D nnU-Net and used cross-entropy, dice coefficient, and boundary loss functions. In the low vertex, adaptive layer-instance normalization (AdaLin) was added to help the attention-guided model correspond to the shape transformation [18]. For learning maxillary sinus segmentation on CBCT, the Adam optimization algorithm with an initial learning rate (3 × 10^−4^) and l2-weight decay (3 × 10^−5^) was used. If the exponential moving average of the training loss did not improve over the previous 30 epochs, the learning rate was reduced by 0.2 times. Training was stopped after exceeding 1000 epochs, or if the learning rate fell below 10^−6^. The analysis of segmentation was calculated using the dice similarity coefficient (DSC), as defined in Equation (1). The loss functions include dice loss (DLS), boundary loss (BLS), and binary cross-entropy (BCE), which are defined in Equations (2)–(4), respectively [19]. $V_{gs}$ is the volume parameter of the ground truth, and $V_{seg}$ is the CNN segmentation.

(1) $$\mathrm{DSC}\;(V_{seg},\;V_{gs},)\;=\;\frac{2\left|V_{seg}\cap \;V_{gs}\right|}{\left|V_{seg}\right|\;+\;\left|V_{gs}\right|},$$

(2) $$\mathrm{DLS}\;=\;1\;-\;\frac{2\left|V_{seg}\cap \;V_{gs}\right|}{\left|V_{seg}\right|\;+\;\left|V_{gs}\right|},$$

(3) $$\mathrm{BLS}(\partial G,\;\partial S)\;=\;2\int_{\Delta S}^{\;}\|q-Z_{\partial }G\left(q\right)\|dq$$

Here, ∆S defines the region between the two contours and ‖*q* − *Z_∂_G*(*q*)‖. Ω → R^+^ is a distance map with respect to boundary *∂G*, that is, ‖*q* − *Z_∂_G*(*q*)‖ evaluates the distance between point q ∈ Ω and the nearest point *Z_∂_G*(*q*) on contour *∂G*: ‖*q* − *Z_∂_G*(*q*)‖.

(4) $$L\;\left(y,\;f\right)\;=\;-y\;\log_{\;}f-\left(1-y\right)\log_{\;}\left(1-f\right)$$

where y is the inferred probability and *f* is the corresponding desired output.

### 2.3. Active Learning

Our active learning framework consists of three steps. This framework adds new volumes per step to improve the performance of the model with limited training datasets, while inferring automatically using the model trained in the previous step.

**Figure 2:**
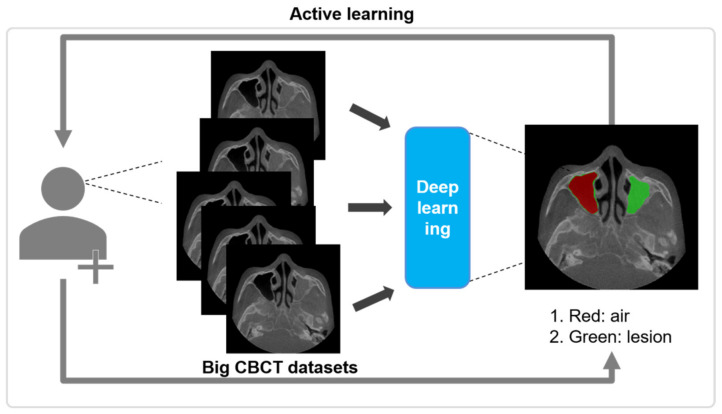
Overall process for the active learning for maxillary sinus segmentation on CBCT.

In the first step, 19 CBCT scans of KUAH were manually labeled by a dentist and an hygienist with more than 7 years of experiences to establish the ground truth. After the labeling process, an oral and maxillofacial surgeon with more than 15 years of experience checked and confirmed all of them. The limited labeled dataset was then initially trained to segment the maxillary sinus on the CBCT of KUAH. After the initial training (first step), the ground truth of the new unlabeled dataset for the next step was acquired for CNN-assisted and post-modified segmentation. In the second step, 19 CBCT scans of KUAH from the first step were reused to train with 30 new datasets, as shown in Figure 2.

After the second step, the CNN-assisted segmentation for the new unlabeled dataset was manually modified for training in the next stage, as performed in the first step. In the final step, 83 scans (49 reused from the second step and 34 new ones) were used to train and improve the model, while the 20 remaining scans (manually labeled in the first step) were used to test each model. The results were evaluated after each step for accurate maxillary sinus segmentation with 20-internal and 20-external scans. The CNN-assisted and post-modified segmentation was conducted using AVIEW Modeler^®^ software, version 1.0.3 (Coreline Software, Seoul, Korea).

In 100 slices (KUAH) and 100 slices (KUANH) selected from internal [air-2633 and lesion-3256 slices] and external [air-3266 and lesion-3988 slices], all manual and the inference of deep learning based on active learning for visual scoring were assessed as very accurate (4 grade) to inaccurate (1 grade).

### 2.4. Experimental Setup

To infer the maxillary sinus on the 3D CBCT volumes of the dental, each axial phase in the volume was inputted sequentially to the model, and multiple 2D segmentation maps were constructed along the z-axis. Only soft tissue lesions such as mucosal thickening or mucosal retention cysts were considered as lesions. The normal soft tissue wall of the maxillary sinus was not considered to be a lesion. The experiment for training and test was conducted on Ubuntu 18.04 with Python 3.6, and used with the TensorFlow 1.15.0 backend with PyTorch 1.4.0 as the deep learning framework. The model was trained on an NVIDIA Titan RTX graphics card (24 GB). To maximize the training speed and optimize the GPU memory, we attempted to use larger input tiles and set the batch size to 6. In the first step, the training saturated approximately after 100 epochs, owing to the small size of the dataset (*n* = 19). The second and last steps required 70 to 100 epochs, owing to the larger datasets (*n* = 49 and *n* = 83). The difference in the overall DSCs between the tuning and test datasets in the final model (step 3) was 2.1. Our model for deep active learning did not overfit for learning with 3D CBCT volumes.

## 3. Results

**Figure 3:**
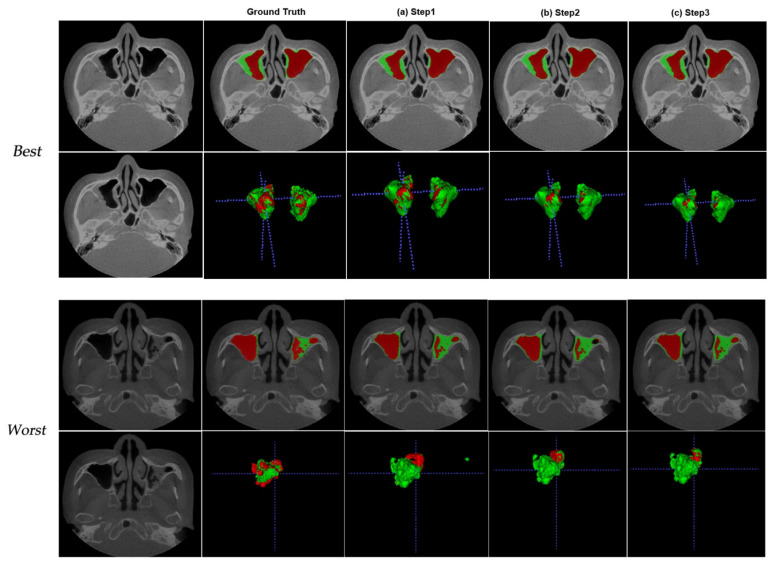
Best (first rows) and worst (second rows) from the test dataset (internal dataset—KUAH) at different analysis points: (**a**) first step, (**b**) second step, and (**c**) last step.

We determined the effect of active learning on CBCT volumes of dental with our customized 3D nnU-Net in all three steps. The DSCs between the ground truth and the prediction were analyzed using 20-internal (KUAH) and 20-external (KUANH) datasets out of the 76 scans that were segmented by active learning. Figure 3 shows the worst and best results for the KUAH. 

The last step is better than the other steps listed in Table 1. The figures show the maxillary sinus segmentation of 3D volumes on CBCT. As the steps progressed, the segmentation results improved on CBCT and reduced the erroneous areas outside the air. The DSCs at each stage of air were 0.920 ± 0.17, 0.925 ± 0.16, and 0.930 ± 0.16, respectively, as shown in Table 2. The DSCs at each stage of the lesion were 0.770 ± 0.18, 0.750 ± 0.19, and 0.760 ± 0.18, respectively (Table 2). 

**Figure 4:**
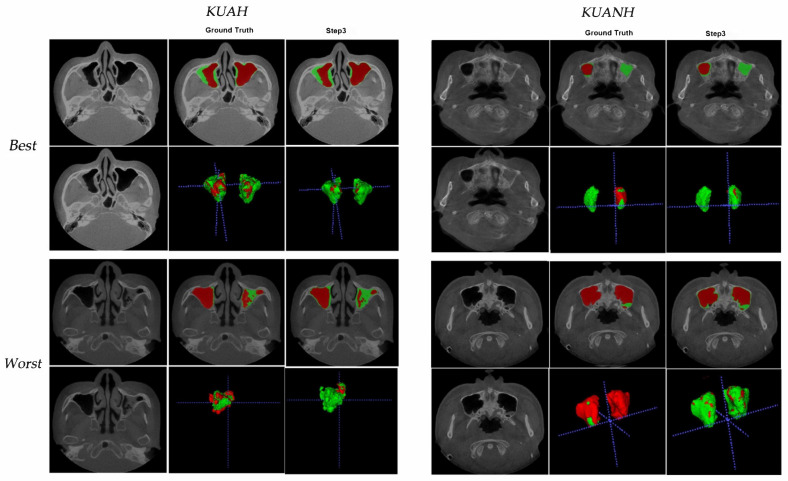
Best (first rows) and worst (second rows) from the test dataset on internal-KUAH and external-KUANH.

The average DSCs for maxillary sinus segmentation increased after each step, and the final segmentation in the last step showed the best results (Table 2). Furthermore, we evaluated the obtained inferences in KUANH using the proposed method. The DSCs for maxillary sinus segmentation in KUANH are presented in Table 3. The results of air on KUANH and KUAH were 0.97 ± 0.02 and 0.93 ± 0.16, respectively. Figure 4 shows the worst and best results for KUAH and KUANH.

Comparisons of the maxillary sinus segmentation times between CNN-assisted and manual segmentation are given in Table 4. The time consumed by the CNN-assisted and manually modified segmentation decreased by approximately 493.2 s for 30 scans in the second step, and by approximately 362.7 s for 76 scans in the last step when compared to that taken in the first step. 

Interestingly, the manual and DL segmentations were classified as very accurate to mostly accurate, and there were few inaccurate cases in Table 5. The number of very accurate cases in the DL segmentations was larger than that for manual segmentations (air: 75.7% vs. 91%, lesion 75% vs. 90%) in KUAH. Most of the slices that indicate DL’s superior performance are seen in the mistakenly drawn manual segmentations of the maxillary sinus area on CBCT.

## 4. Discussion

In this study, we proposed an active learning framework for maxillary sinus segmentation using a customized 3D nnU-Net on CBCT [16]. The most difficult part of maxillary sinus segmentation was separation, with the opening part that connects with other areas. In particular, the ethmoidal area was difficult because there were many open areas with several small holes. In addition, the part that connects to the nasal cavity is also large, so it is not easy to separate. Discriminating whether it is the maxillary sinus, nasal cavity, or ethmoidal region is a task requiring considerable difficulty even for a specialist. The value of this study lies in the development of a technology that can easily separate maxillary sinus lesions with the help of artificial intelligence.

In addition, the performance of artificial intelligence models has been improved using active learning [20]. As each step was completed, the DSCs increased and exhibited excellent performance. As shown in Table 4, the labeling time for CNN-assisted segmentation is reduced by more than half compared to manual segmentation. Segmentation accuracy increased over the steps, and the overall performance was reasonable compared with other state-of-the-art segmentation networks. 3D segmentation of the maxillary sinus is not an easy task. External validation was performed by dental specialists at both hospitals. No matter how accurately the segmentation was performed, a slight error in the boundary area inevitably occurs when a person performs it manually. CNN segmentation by AI first and modification is more efficient and time-saving compared to manual labeling from scratch. Therefore, it can be concluded that active learning can reduce the labeling effort through CNN-assisted segmentation and increase training efficiency through iterative learning with limited data.

When comparing its performance with other similar studies, 3D U-Net has become one of the most popular methods for pixel-by-pixel semantic segmentation because it shows excellent performance in medical image processing. However, several researchers have further advanced this network by combining detection architectures and cascading methods. Tang et al. proposed a cascade framework consisting of a detection architecture and a segmentation module using the VGG-16 model [21]. Roth et al. also proposed a two-stage FCN model in a cascading manner, with a focus on the target boundary area [22]. Yang et al. proposed a deep active learning framework by combining an FCN with active learning [23]. Lubrano et al. also proposed a similar framework for the segmentation of myelin sheaths in histological data [24]. The authors used Monte Carlo dropout to evaluate the model uncertainty and select samples for labeling. The segmentation performance of this study showed similar or better performance by showing DSC values of 0.92~0.93 in the air layer and 0.75~0.77 in the lesion compared to other studies showing DSC values of 0.66~0.85 [5,25,26,27]. In addition, unlike other studies, in this study, because the test was performed with multi-center data, it can be said that the generalization of performance was further verified.

In this study, the results of lesion segmentation were inferior to those of air segmentation in this study. Air with a certain radiopacity can be easily separated through threshold adjustment, while the separation of lesions with various radiopacities is difficult. To label the lesion, the entire maxillary sinus area was separated first, and the air layer was excluded. In the process of separating the maxillary sinus, a part of the bone was also included, and errors could also occur in the process of separating it from the adjacent sinuses. In addition, the thickness of the soft tissue wall surrounding the maxillary sinus varies from person to person. In the case of a thin soft tissue wall, a break may occur during the separation process, so the lesion may not be separated neatly.

The limitation of this study is that the segmentation performance of the maxillary sinus appeared to be low when the entire maxillary sinus was filled with inflammatory material. In the case of severe maxillary sinusitis, it can be seen that the inside of the maxillary sinus is hazy, which can be seen as a case where water or inflammatory substances are filled in the maxillary sinus. These artifacts are a factor that makes segmentation difficult. To overcome this, further studies are needed to increase our training dataset and use a better network to address ambiguities to improve segmentation performance. To improve the effectiveness and accuracy of the proposed scheme, further validation with more multi-center datasets and comparisons with other segmented networks, such as cascade networks, should be performed.

As labeling is basic but very labor-intensive, active learning can be considered to be a useful alternative [28,29]. In addition, manual labeling is not always constant in the segmentation process because of differences between people. Active learning frameworks can reduce this uncertainty by improving the accuracy by increasing collaboration with deep-learning algorithms. In addition, it is necessary to study the classification of segmented lesions in the future. This study suggests that, even for organs with complex structures such as the skull, the use of segmentation, lesion analysis, and diagnosis using active learning can be widely used.

## 5. Conclusions

In conclusion, this study demonstrates that a deep active learning framework (human-in-the-loop) can alleviate annotation efforts and costs by efficiently training limited CBCT datasets.

## Figures and Tables

**Table 1:** Characteristics and acquisition parameters of the study population by group.

Characteristic	Training and Tuning (KUAH) (*n* = 83)	Internal-Validation (KUAH) (*n* = 20)	External-Validation KUANH (*n* = 20)
Age	59.9 ± 17.2	63.1 ± 16.9	40 ± 19.7
Male	44	10	10
Female	39	10	10
Tube voltage (kV)	120	120	90
Tube current (mA)	5	5	4
Scan time (s)	16.8	16.8	14.3
Voxel size (mm)	0.3	0.3	0.3
FOV (mm)	230 × 170	230 × 170	170 × 135
Focal spot (mm)	0.58	0.58	0.70

Note: Internal dataset: Korea University Anam Hospital (KUAH); external dataset—Korea University Ansan Hospital (KUANH); Field-of-view (FOV).

**Table 2:** DSCs for the first, second, and last steps for the test dataset (20 cases) on KUAH.

Mean ± SD (Range)	First Step	Second Step	Last Step
Air	0.920 ± 0.17 (0.245–0.992)	0.925 ± 0.16 (0.241–0.991)	0.930 ± 0.16 (0.243–0.996)
Lesion	0.770 ± 0.18 (0.208–0.912)	0.750 ± 0.19 (0.205–0.975)	0.760 ± 0.18 (0.208–0.96)

Note: Dice similarity coefficient (DSC); Korea University Anam Hospital (KUAH); Standard deviation (SD).

**Table 3:** DSCs for the test dataset (20 cases of internal-KUAH and 20 cases of external-KUANH) in Figure 1; 3D-nnU-Net.

Mean ± SD (Range)	Last step (KUAH)	Last step (KUANH)
Air	0.93 ± 0.16 (0.243–0.996)	0.97 ± 0.02 (0.94–0.99)
Lesion	0.76 ± 0.18 (0.208–0.96)	0.54 ± 0.23 (0.12–0.88)

Note: Dice similarity coefficient (DSC); Korea University Anam Hospital (KUAH); Korea University Ansan Hospital (KUANH); Standard deviation (SD).

**Table 4:** Comparison of segmentation times between the manual and CNN-assisted and manually modified segmentation approaches.

	First Step	Second Step	Last Step
	Manual segmentation	CNN-assisted and manually modified segmentation	CNN-assisted and manually modified segmentation
Time	1824.0 s	493.2 s	362.7 s

Note: Convolutional neural network (CNN).

**Table 5:** Qualitative results from visual scoring of automatic maxillary sinus segmentation on CBCT from 100 Randomly Selected slices (internal-KUAH and external-KUANH) *.

Grade	Manual				3D U-Net (Last Step for Active Learning)			
	KUAH		KUANH		KUAH		KUANH	
	Air	Lesion	Air	Lesion	Air	Lesion	Air	Lesion
4—Very accurate	75.7	75	83.7	79.7	91	90	95.3	88
3—Accurate	19.6	16.6	15.3	19.3	8	7.4	4.7	12
2—Mostly accurate	1	3.7	1	1	0	2.3	0	0
1—Inaccurate	3.7	4.7	0	0	0	0.3	0	0

Note: Korea University Anam Hospital (KUAH); Korea University Ansan Hospital (KUANH); * Four-point scale: Three dentists conducted grade (manual vs. deep learning). 4—Very accurate: when the labelled maxillary sinus part completely matches the original sinus (over 95%); 3—Accurate: when the labelled sinus almost completely matches the original maxillary sinus (85–95%); 2—Mostly accurate: when the labelled maxillary sinus part depicts the site of the original maxillary sinus area (over 50%); 1—Inaccurate: when the labelled part depicts outside of the sinus or only matches small area of original maxillary sinus (under 50%).

## Data Availability

The data underlying this article will be shared on reasonable request from the corresponding author.
